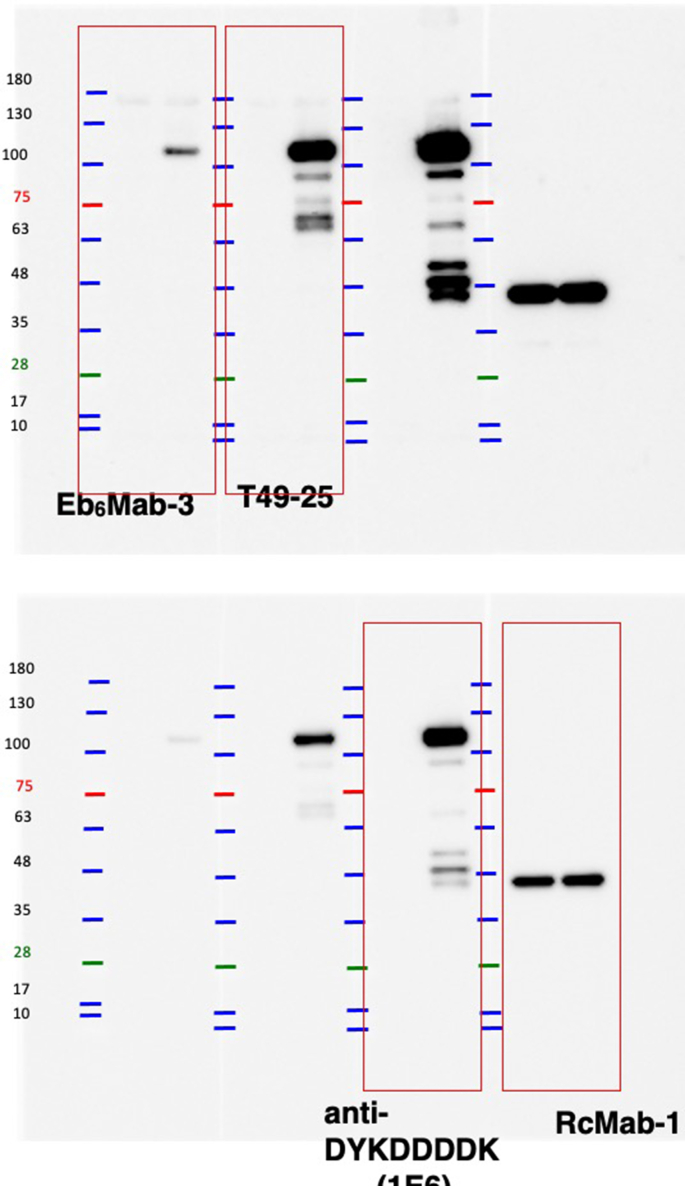# Development of a novel anti-erythropoietin-producing hepatocellular receptor B6 monoclonal antibody Eb_6_Mab-3 for flow cytometry

**DOI:** 10.1016/j.bbrep.2025.101960

**Published:** 2025-02-21

**Authors:** Tomohiro Tanaka, Yu Kaneko, Haruto Yamamoto, Guanjie Li, Shiori Fujisawa, Hiroyuki Satofuka, Keisuke Shinoda, Takuya Nakamura, Mika K. Kaneko, Hiroyuki Suzuki, Yukinari Kato

**Affiliations:** Department of Antibody Drug Development, Tohoku University Graduate School of Medicine, 2-1, Seiryo-machi, Aoba-ku, Sendai, Miyagi, 980-8575, Japan

**Keywords:** Eph receptor, EphB6, CBIS method, Monoclonal antibody, Flow cytometry

## Abstract

Erythropoietin-producing hepatocellular receptor B6 (EphB6) is a member of the largest Eph subfamily of receptor tyrosine kinases. EphB6 is widely expressed in various tissues and regulates cellular homeostasis by interacting with its membrane-bound ephrin ligands and other receptors. EphB6 is involved in cancer pathology despite lacking kinase activity. Developing sensitive monoclonal antibodies (mAbs) for EphB6 has been desired for treatment, diagnosis, and further analysis of EphB6. This study established a novel specific and sensitive anti-human EphB6 mAb clone Eb_6_Mab-3 (mouse IgG_1_, kappa) by the Cell-Based Immunization and Screening (CBIS) method. In flow cytometry, Eb_6_Mab-3 demonstrated reactivity with EphB6-overexpressed Chinese hamster ovary-K1 cells (CHO/EphB6) and endogenously EphB6-expressing DLD-1 colorectal cancer cells. Cross-reactivity of Eb_6_Mab-3 was not observed. Eb_6_Mab-3 demonstrated a moderate binding affinity (dissociation constant; *K*_D_) for CHO/EphB6 (*K*_D_: 2.6 ± 1.0 × 10^−8^ M) and a high binding affinity for DLD-1 (*K*_D_: 3.4 ± 1.3 × 10^−9^ M). Eb_6_Mab-3 can detect EphB6 protein in CHO/EphB6 lysate in Western blot. Eb_6_Mab-3, established by the CBIS method, could be valuable for analyzing the EphB6-associated cellular functions and has potential applications in diagnosis and treatment with specificity and high affinity for cancer cells.

## Highlights

• A novel anti-EphB6 monoclonal antibody, Eb_6_Mab-3 was developed by the Cell-Based Immunization and Screening method.
• Eb_6_Mab-3 is useful for the detection of EphB6 by flow cytometry and western blot analyses.
• Eb_6_Mab-3 can detect EphB6-positive cells with high affinity and specificity.

## 1 Introduction

Receptor tyrosine kinases (RTKs) play pivotal roles in tissue homeostasis, including cell proliferation, differentiation, migration, tissue remodeling, and angiogenesis [[1], [2], [3], [4], [5]]. Aberrant signaling of abnormal RTKs leads to disordered cellular homeostasis and tumor development [6,7]. Erythropoietin-producing hepatocellular (Eph) receptors belong to the most prominent family of RTKs and exert diverse functions by binding to their ligands, ephrin. The Eph and ephrin families comprise 14 Eph receptors (EphA1 to EphA8, EphA10, EphB1 to EphB4, and EphB6) and eight ephrin ligands [glycosylphosphatidylinositol (GPI)-anchored ephrin A1 to A5 and transmembrane ephrin B1 to B3], respectively [8]. The intercellular Eph and ephrin dimerization and clustering mediate forward and reverse signaling, respectively [9,10]. Eph receptors and ephrin can affect various cell types in healthy tissues and disorders through these signaling [[11], [12], [13]].

EphB6 and EphA10 are pseudokinases that contain pseudokinase domain lacking kinase activity in the intracellular domain [[14], [15], [16]]. The ligands for EphB6 are still unclear, but ephrinB1 and ephrinB2 are the candidates [17,18]. Despite inactive kinase, EphB6 plays a vital role in controlling the cell functions by binding the ephrins and interacting with other RTKs. EphB6 serves as an oncogene which involved in the development and malignancy of tumors, including colon [19], leukemia [20,21], tongue squamous cell carcinoma [22], breast [23], bladder [24], and non-small cell lung cancer (NSCLC) [25]. EphB6 undergoes tyrosine transphosphorylation upon stimulation with ephrin B1 and ephrin B2 by forming a cluster with EphB1 and EphB4 [26,27]. Also, the interaction of EphB6 and EphA2 has been observed in breast cancer cell lines [28]. These findings suggest that EphB6 possesses valuable functions even in a kinase-dead RTK by cross-talking with kinase-active partners and might contribute to cancer progression. In clinical thyroid malignant lesions from patients, the higher expression of EphB6 and EphB4 has been observed compared to benign ones and correlated with tumor size [29].

Interestingly, numerous reports have also described the role of EphB6 as a tumor suppressor. Loss of EphB6 brings tumor malignancy and poor prognosis [23,[30], [31], [32], [33], [34]]. Low EphB6 expression is associated with poor TNM stage and tumor grade in ovarian serous carcinoma and neuroblastoma [35,36]. Also, in molecular level analysis, EphB6 suppresses EphA2-promoted anoikis of breast cancer cells by interfering with EphA2-Ephexin 4 interaction [28]. In EphB6 null mice, the T cells had reduced the secretion of interleukin-2 (IL-2), IL-4, and interferon-γ [37]. Conversely, T cell proliferation and lymphokines secretion are enhanced by co-stimulation of EphB6 and T cell receptor [38]. These reports suggest that EphB6 exhibits tumor-suppressive functions within cancer cells and through the immune systems.

Mutations in the EphB6 gene that promote tumor metastasis have been identified in NSCLC patients [25]. The EphB6 mutation mediates paclitaxel resistance by simultaneously upregulating the expression of EphA2 and cadherin 11 [39]. Further analysis is essential to determine whether EphB6 promotes or suppresses tumors, and the development of highly sensitive antibodies against EphB6 is desired for basic research, diagnosis, and treatment.

Drugs specifically targeting Eph receptors or ephrin ligands have yet to be approved. However, Eph receptors have been widely studied in relation to cancer and considered therapeutic targets [8]. Regarding antibody drugs, the Phase I trial of DS-8895a, a humanized anti-EphA2 defucosylated mAb, has been performed against advanced EphA2-expressing cancer [40,41]. Ifabotuzumab (KB004), an anti-EphA3 mAb, has been tested in advanced hematologic malignancies [42].

Previously, we have established monoclonal antibodies (mAbs) against human EphA2 (clone Ea_2_Mab-7) [43], human EphB2 (clone Eb_2_Mab-3) [44], and human EphB4 (clone B4Mab-7) [45] by using the Cell-Based Immunization and Screening (CBIS) method. This method can efficiently develop a wide variety of antibodies that recognize linear epitope, structural epitope, and modifications of extracellular domains of membrane protein in a short period. In this study, we have successfully established a novel anti-human EphB6 mAb (clone Eb_6_Mab-3) using the CBIS method.

## 2 Materials and methods

### 2.1 Cell lines

Cell lines, including LN229, Chinese hamster ovary (CHO)–K1, and P3X63Ag8U.1 (P3U1) cells were obtained from the American Type Culture Collection (Manassas, VA, USA). DLD-1 cells were obtained from the Cell Resource Center for Biomedical Research, Institute of Development, Aging and Cancer, Tohoku University (Miyagi, Japan). The expression plasmid of EphB6 (pCMV6neoEphB6-Myc-DDK, Catalog No.: RC229404, Accession No.: NM_004445, OriGene Technologies, Inc. Rockville, MD, USA) was transfected into cell lines using the Neon transfection system (Thermo Fisher Scientific, Inc., Waltham, MA, USA). Subsequently, LN229 and CHO–K1, which stably overexpressed EphB6 with C-terminal Myc-DDK tags (hereafter described as LN229/EphB6 and CHO/EphB6, respectively) were stained with an anti-EphB6 mAb (clone T49-25; BioLegend, San Diego, CA, USA) and sorted using the SH800 cell sorter (Sony corp., Tokyo, Japan), followed by cultivation in a medium containing 0.5 mg/mL of G418 (Nacalai Tesque, Inc., Kyoto, Japan).

The complementary DNAs (cDNAs) of other Eph receptors, including EphA1 (Catalog No.: RC213689, Accession No.: NM_005232), EphA4 (Catalog No.: RC211230, Accession No.: NM_004438), EphA5 (Catalog No.: RC213206, Accession No.: NM_004439), EphA6 (Catalog No.: RC223510, Accession No.: NM_001080448), EphA7 (Catalog No.: RC226293, Accession No.: NM_004440), EphA8 (Accession No. NM_020526; Catalog No.: RC220352), EphA10 (Catalog No.: RC218374, Accession No.: NM_001099439), EphB1 (Catalog No.: RC214301, Accession No.: NM_004441), EphB2 (Catalog No.: RC223882, Accession No.: NM_004442) were purchased from OriGene Technologies (Rockville, MD, USA), Inc. EphA2 (Catalog No.: HGY095959, Accession No.: NM_004431), EphA3 (Catalog No.: HGY053437, Accession No.: NM_005233), and EphB3 (Catalog No.: HGX039581, Accession No.: NM_004443) cDNAs were purchased from RIKEN DNA Bank (Ibaraki, Japan).

EphA2 and EphB3 cDNAs were cloned into a pCAGzeo vector [FUJIFILM Wako Pure Chemical Corporation (Wako), Osaka, Japan]. EphA1 cDNA was cloned into a pCAGzeo-ssnPA vector. EphA3, EphA4, EphA5, EphA6, EphA7, EphA8, EphA10, and EphB1 cDNA were cloned into a pCAGzeo_ssnPA16 vector.

The plasmids were also transfected into CHO–K1 cells and stable transfectants were established by staining with an anti-EphA2 mAb (clone SHM16; BioLegend), an anti-EphB2 mAb (clone 2H9; BD Bioscience, Franklin Lakes, NJ, USA), an anti-EphB3 mAb (clone 647354; R&D Systems Inc., Minneapolis, MN, USA), and an anti-PA tag [46] mAb (clone NZ-1 for EphA1, EphA3, EphA4, EphA5, EphA6, EphA7, EphA8, EphA10, and EphB1), and sorted using SH800. After sorting, cultivation in a medium containing 0.5 mg/mL of Zeocin (InvivoGen, San Diego, CA, USA) or 0.5 mg/mL of G418 was progressed. These Eph receptors-overexpressed CHO–K1 (e.g., CHO/EphA1) clones were finally established. CHO/PA16-EphB4 was previously described [45].

CHO–K1, P3U1, Eph receptor-overexpressed CHO–K1, and DLD-1 cells were also cultured in a Roswell Park Memorial Institute (RPMI)-1640 medium (Nacalai Tesque, Inc.) that was supplemented with 10 % heat-inactivated fetal bovine serum (FBS, Thermo Fisher Scientific Inc.), 100 units/mL penicillin, 100 μg/mL streptomycin, and 0.25 μg/mL amphotericin B (Nacalai Tesque, Inc.). LN229 and LN229/EphB6 were cultured in a Dulbecco's Modified Eagle Medium (DMEM) (Nacalai Tesque, Inc.) that was supplemented with 10 % heat-inactivated FBS (Thermo Fisher Scientific Inc.), 100 units/mL penicillin, 100 μg/mL streptomycin, and 0.25 μg/mL amphotericin B (Nacalai Tesque, Inc.). Then, cells were cultured in a humidified CO_2_ incubator with 5 % CO_2_ and 95 % air at 37 °C.

### 2.2 Antibodies

An anti-human EPHB6 mAb (clone T49-25, mouse IgG_1_, kappa) was purchased from BioLegend. An anti-DYKDDDDK (clone 1E6) mAb was purchased from Wako. An anti-isocitrate dehydrogenase 1 (IDH1) mAb (clone RcMab-1) was developed previously in our lab [47]. A secondary Alexa Fluor 488-conjugated anti-mouse IgG was purchased from Cell Signaling Technology, Inc. (Danvers, MA, USA). Secondary horseradish peroxidase-conjugated anti-mouse IgG and anti-rat IgG were obtained from Agilent Technologies Inc. (Santa Clara, CA, USA) and Merck KGaA (Darmstadt, Germany), respectively.

### 2.3 Hybridoma production

For developing anti-EphB6 mAbs, two 6-week-old female BALB/cAJcl mice, purchased from CLEA Japan (Tokyo, Japan), were immunized intraperitoneally with 1 × 10^8^ cells/mouse of LN229/EphB6. The LN229/EphB6 cells as immunogen were harvested after brief exposure to 1 mM ethylenediaminetetraacetic acid (EDTA; Nacalai Tesque, Inc.). Alhydrogel adjuvant 2 % (InvivoGen, San Diego, CA, USA) was added as an adjuvant in the first immunization. Three additional injections of 1 × 10^8^ cells/mouse of LN229/EphB6 were administered intraperitoneally without an adjuvant addition every week. A final booster injection was performed with 1 × 10^8^ cells/mouse of LN229/EphB6 intraperitoneally two days before harvesting splenocytes from mice. We conducted cell-fusion of the harvested splenocytes from immunized mice with P3U1 cells using polyethylene glycol 1500 (PEG1500; Roche Diagnostics, Indianapolis, IN, USA) under heated conditions.

Hybridomas were cultured in the RPMI-1640 medium supplemented as shown above, with additional supplements included hypoxanthine, aminopterin, and thymidine (HAT; Thermo Fisher Scientific, Inc.), 5 % BriClone (NICB, Dublin, Ireland), and 5 μg/mL of Plasmocin (InvivoGen) into the medium. The hybridoma supernatants were screened by flow cytometry using CHO/EphB6 and parental CHO–K1 cells. The hybridoma supernatant, containing Eb_6_Mab-3 in serum free-medium, was filtrated and purified using Ab-Capcher Extra (ProteNova, Kagawa, Japan).

### 2.4 Flow cytometry

Cells were harvested using 0.25 % trypsin and 1 mM ethylenediaminetetraacetic acid (EDTA; Nacalai Tesque, Inc.) or 1 mM EDTA. Subsequently, cells were washed with 0.1 % bovine serum albumin in phosphate-buffered saline (PBS) and treated with primary mAbs for 30 min at 4 °C. Afterward, cells were treated with Alexa Fluor 488-conjugated anti-mouse IgG (1:1000) following the collection of fluorescence data using the SA3800 Cell Analyzer (Sony Corp.).

### 2.5 Determination of the binding affinity by flow cytometry

CHO/EphB6 and DLD-1 cells were suspended in 100 μL serially diluted Eb_6_Mab-3 (50 μg/mL to 0.003 μg/mL for CHO/EphB6, 100 μg/mL to 0.006 μg/mL for DLD-1) and T49-25 (50 μg/mL to 0.003 μg/mL) after which Alexa Fluor 488-conjugated anti-mouse IgG (1:200) was added. Fluorescence data were subsequently collected using the SA3800 Cell Analyzer, following the calculation of the dissociation constant (*K*_D_) by fitting the binding isotherms into the built-in one-site binding model in GraphPad PRISM 6 (GraphPad Software, Inc., La Jolla, CA, USA).

### 2.6 Western blot analysis

Cell lysates (10 μg/lane) were boiled in sodium dodecyl sulfate (SDS) sample buffer (Nacalai Tesque, Inc.). Proteins were electrophoresed on 5%–20 % polyacrylamide gels (Wako) and transferred onto polyvinylidene difluoride (PVDF) membranes (Merck KGaA). After blocking with 4 % non-fat milk (Nacalai Tesque, Inc.), PVDF membranes were incubated with 5 μg/mL of Eb_6_Mab-3, 2.5 μg/mL of T49-25, 1 μg/mL of an anti-IDH1 mAb (clone RcMab-1), or 0.5 μg/mL anti-DYKDDDDK (clone 1E6, Wako) mAb, followed by incubation with horseradish peroxidase-conjugated anti-mouse IgG (1:2000; Agilent Technologies Inc.) or anti-rat IgG (1:10000; Merck KGaA). Chemiluminescence signals were developed using ImmunoStar LD (Wako) and imaged with a Sayaca-Imager (DRC Co. Ltd., Tokyo, Japan).

## 3 Results

### 3.1 Development of anti-EphB6 mAbs using the CBIS method

**Fig. 1:**
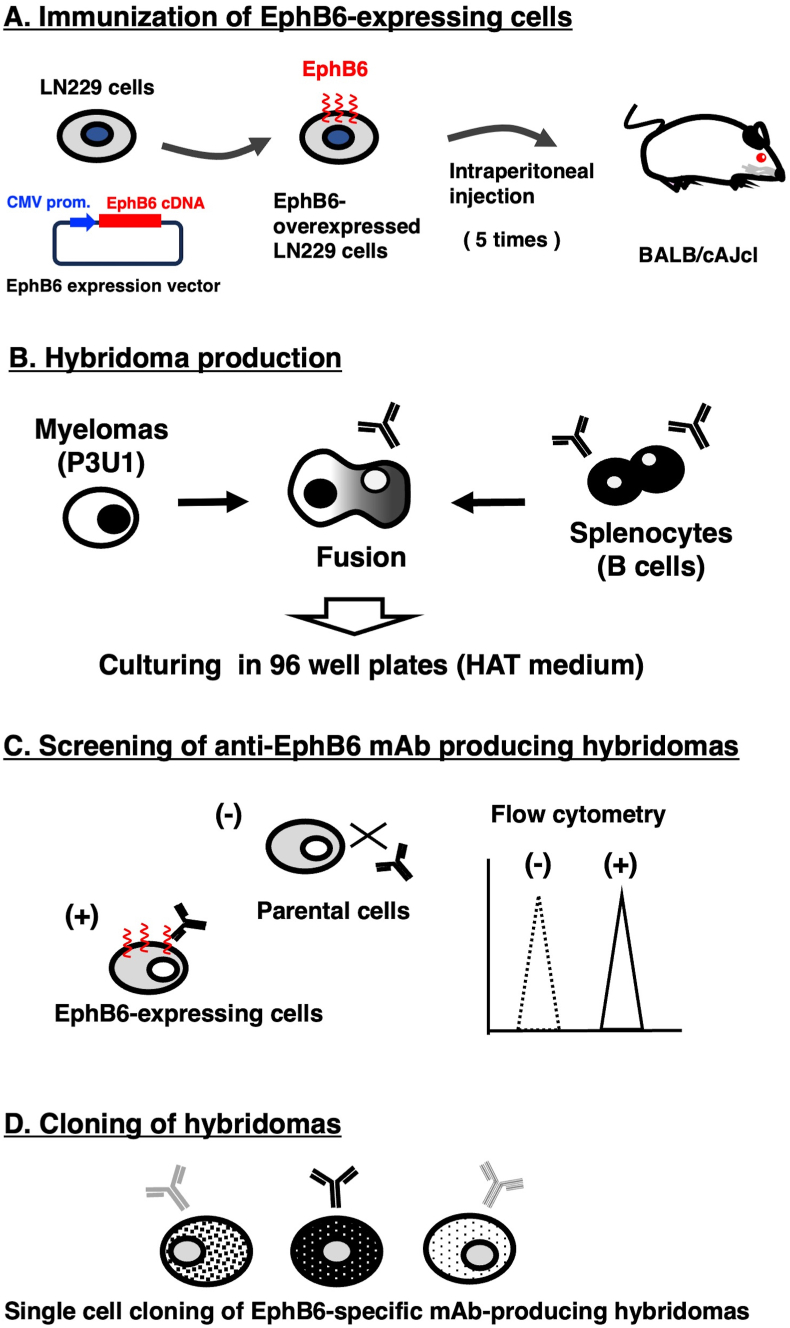
A schematic depiction of anti-EphB6 mAbs development by CBIS method. The simplified procedure of antibody development using the CBIS method. (A) LN229/EphB6 cells were intraperitoneally immunized into two female mice. (B) The spleen cells from antigen-immunized mice were fused with myeloma cells, P3U1, by PEG1500. (C) The culture supernatants of hybridoma were screened by flow cytometry using CHO–K1 and CHO/EphB6 to select EphB6-specific mAb-producing hybridomas. (D) Single hybridoma clones were obtained by limiting dilution, followed by additional screening. Finally, Eb_6_Mab-3 (mouse IgG_1_, kappa) was established.

To establish anti-EphB6 mAbs, we employed the CBIS method using EphB6-overexpressed cells. Anti-EphB6 mAbs-producing hybridomas were screened by using flow cytometry (Fig. 1). Two female BALB/cAJcl mice were intraperitoneally immunized with LN229/EphB6 (1 × 10^8^ cells/time/mouse) every week, a total of 5 times. Subsequently, mouse splenocytes and P3U1 cells were fused by PEG1500. Hybridomas were seeded into 96-well plates, after which the flow cytometric screening was conducted to select CHO/EphB6-reactive and parental CHO–K1-nonreactive supernatants of hybridomas. We obtained some highly CHO/EphB6-reactive supernatants of hybridomas. We finally established the highly sensitive clone Eb_6_Mab-3 (mouse IgG_1_, kappa) by limiting dilution and additional analysis.

### 3.2 Evaluation of antibody reactivity using flow cytometry

**Fig. 2:**
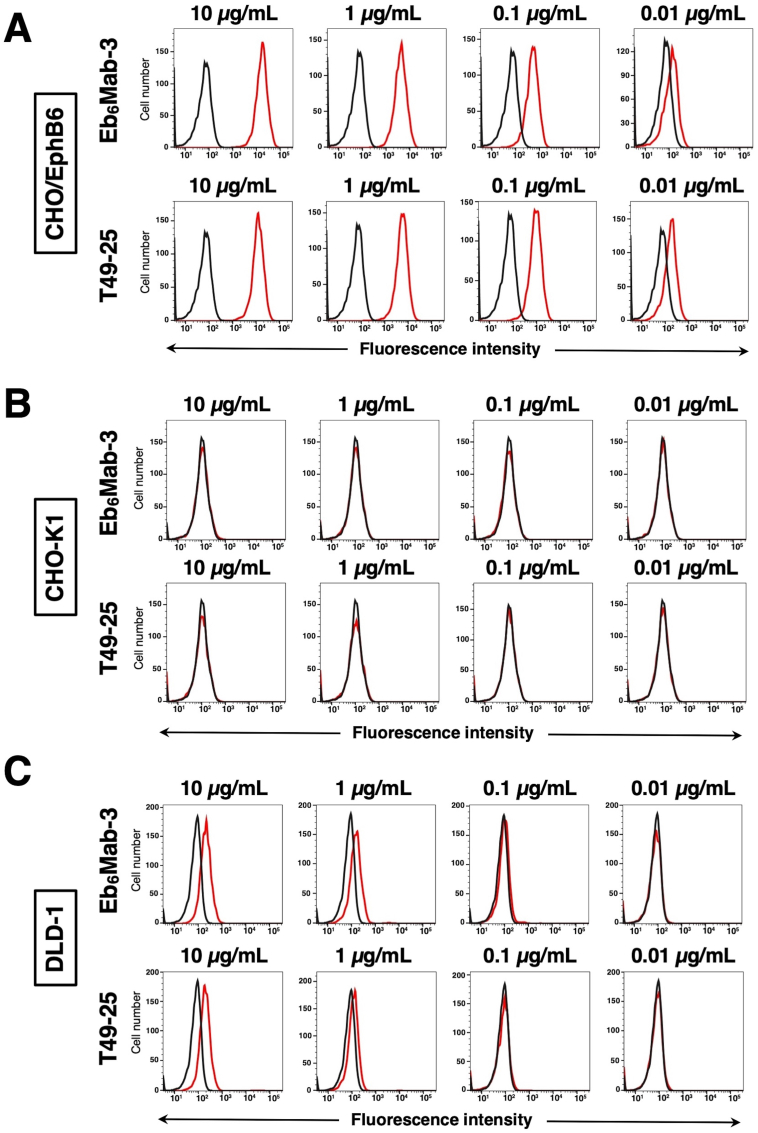
Flow cytometric analysis of anti-EphB6 mAbs. CHO/EphB6 (A), CHO–K1 (B), and DLD-1 (C) cells were treated with 0.01–10 μg/mL of Eb_6_Mab-3 or T49-25 (red line), followed by treatment with Alexa Fluor 488-conjugated anti-mouse IgG. Fluorescence data were collected using the SA3800 Cell Analyzer. Black line, control (no primary antibody treatment).

Flow cytometric analysis was conducted using Eb_6_Mab-3 and commercially available anti-EphB6 mAb (T49-25) against CHO–K1, CHO/EphB6, and DLD-1 cells. EphB6 is frequently expressed in colorectal cancer [19]. Results indicated that Eb_6_Mab-3 and T49-25 recognized CHO/EphB6 (Fig. 2A) dose-dependently. Reactivity is almost the same between Eb_6_Mab-3 and T49-25 to CHO/EphB6 (Fig. 2A). Neither Eb_6_Mab-3 nor T49-25 reacted with parental CHO–K1 cells even at a concentration of 10 μg/mL (Fig. 2B). Eb_6_Mab-3 showed slightly higher reactivity than T49-25 at 1 μg/mL of mAbs to DLD-1 (Fig. 2C). The reactivity was saturated at more than 10 μg/mL (Fig. S1). Thus, Eb_6_Mab-3 can detect exogenously and endogenously expressing EphB6 in flow cytometry.

### 3.3 Specificity of Eb_6_Mab-3 to Eph receptor-overexpressed CHO–K1 cells

**Fig. 3:**
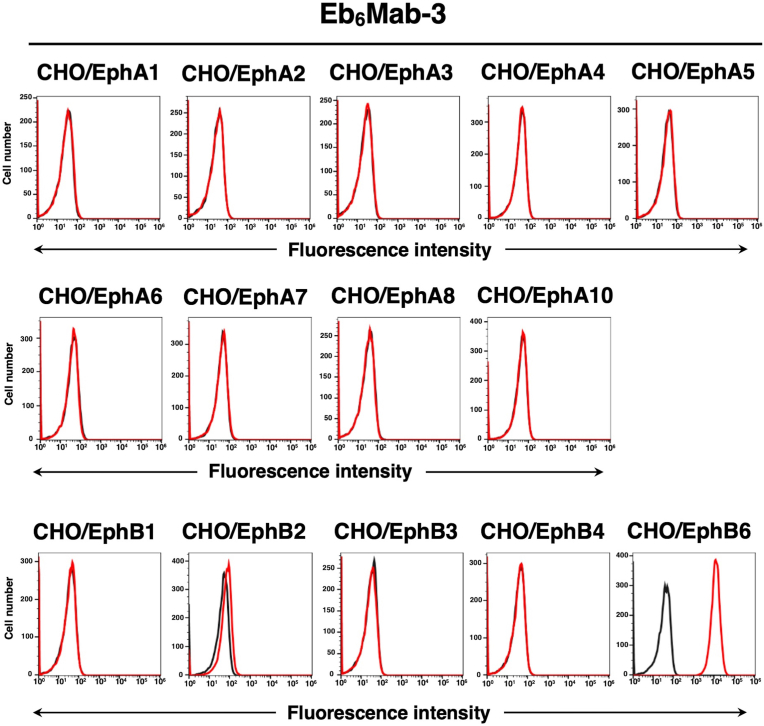
Flow cytometry of Eb_6_Mab-3 in Eph receptor-expressed CHO–K1 cells. CHO–K1 cells which overexpressed each of the fourteen Eph receptors [48] were treated with 10 μg/mL of Eb_6_Mab-3 (red line) or control blocking buffer (black line) followed by the treatment with anti-mouse IgG conjugated with Alexa Fluor 488.

We have established the cell lines of all Eph receptor-overexpressed CHO–K1 cells, EphA1 to A8, A10, B1 to B4, and B6, respectively. We previously reported the cell surface expression of Eph receptors using flow cytometry [48]. Using the 14 cell lines, the specificity of Eb_6_Mab-3 was analyzed. As shown in Fig. 3, 10 μg/mL of Eb_6_Mab-3 potently recognized CHO/EphB6. Weak recognition to CHO/EphB2 by Eb_6_Mab-3 was observed.

### 3.4 Calculation of the binding affinity of anti-EphB6 mAbs using flow cytometry

**Fig. 4:**
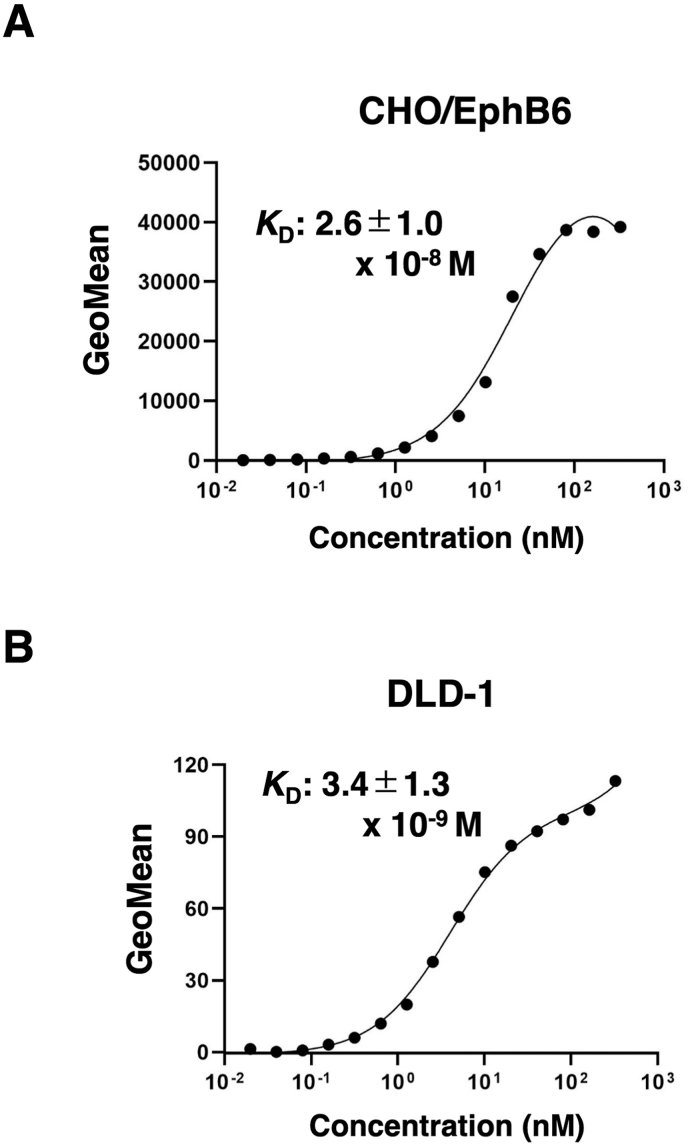
Determination of the binding affinity of Eb_6_Mab-3. CHO/EphB6 (A) and DLD-1 (B) cells were suspended in 100 μL of serially diluted 50 μg/mL to 0.003 μg/mL of Eb_6_Mab-3 for CHO/EphB6, 100 μg/mL to 0.006 μg/mL of Eb_6_Mab-3 for DLD-1. Then, cells were treated with Alexa Fluor 488-conjugated anti-mouse IgG. Subsequently, the geometric mean values from fluorescence data were determined using the SA3800 Cell Analyzer. The average *K*_D_ values (± *standard deviation*) from three independent measurements were calculated by GraphPad PRISM 6 software. The representative graphs were shown.

The binding affinity of Eb_6_Mab-3 and T49-25 were assessed with exogenously EphB6-expressed CHO/EphB6 and endogenously EphB6-expressing DLD-1 using flow cytometry. From three independent measurement (Fig. S2), *K*_D_ values of Eb_6_Mab-3 for CHO/EphB6 and DLD-1 were determined as (2.6 ± 1.0) × 10^−8^ M and (3.4 ± 1.3) × 10^−9^ M, respectively (Fig. 4). The *K*_D_ values of T49-25 for CHO/EphB6 and DLD-1 were 1.5 × 10^−8^ M and 1.3 × 10^−8^ M, respectively (Fig. S3). Although there was no noticeable difference in binding affinity for CHO/EphB6 between Eb_6_Mab-3 and T49-25, Eb_6_Mab-3 showed higher binding affinity for DLD-1 than T49-25. These results demonstrate that Eb_6_Mab-3 can recognize EphB6 with moderate to high affinity to EphB6 on cells.

### 3.5 Western blot analysis using anti-EphB6 mAbs

**Fig. 5:**
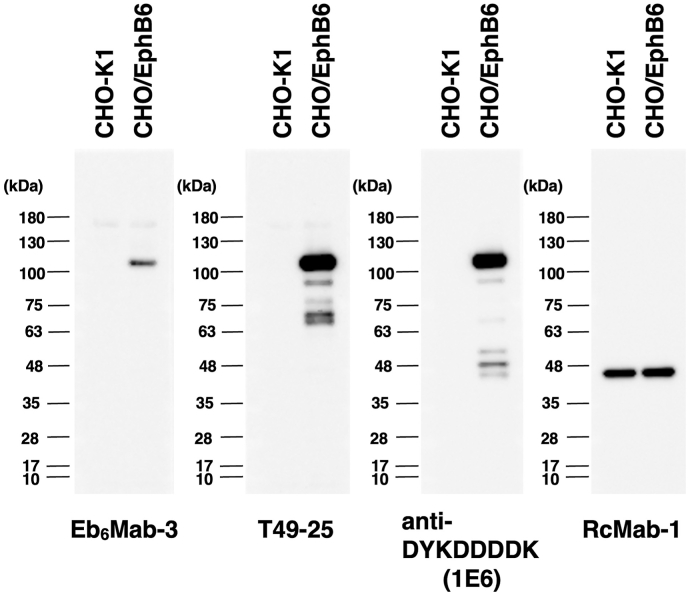
The detection of EphB6 by Western blot analysis. Cell lysates of CHO–K1 and CHO/EphB6 (10 μg/lane) were electrophoresed and transferred onto PVDF membranes. The membranes were incubated with 5 μg/mL of Eb_6_Mab-3, 2.5 μg/mL of T49-25, 0.5 μg/mL of an anti-DYKDDDDK (clone 1E6) mAb, and 1 μg/mL of RcMab-1 and subsequently with horseradish peroxidase-conjugated anti-mouse or anti-rat immunoglobulins.

We investigated whether Eb_6_Mab-3 can be used for Western blot analysis by analyzing CHO–K1 and EphB6-overexpressed CHO/EphB6 cell lysates. The estimated molecular weight of EphB6 protein is 110-kDa. As shown in Fig. 5, Eb_6_Mab-3 could detect EphB6 as the band around 100 to 130-kDa (EphB6 + Myc-DDK tags) in CHO/EphB6 cell lysates, while no band was detected in parental CHO–K1 cells. Another anti-EphB6 mAb (clone T49-25) could clearly detect EphB6 as the band around 100 to 130-kDa in CHO/EphB6 cell lysates. In Western blot, T49-25 appears to detect EphB6 more sensitively than Eb_6_Mab-3. An anti-DYKDDDDK mAb was used as a positive control and could detect a band of the same position in CHO/EphB6 cell lysates. An anti-IDH1 mAb (clone RcMab-1) was used for internal control. The CBB staining of the gel is shown in Fig. S4. These results indicate that Eb_6_Mab-3 can detect EphB6 in EphB6-overexpressing cells in Western blot analysis.

Further investigation was conducted to explore other applications, such as immunohistochemistry. Unfortunately, Eb_6_Mab-3 could not detect EphB6 by immunohistochemistry using cell blocks of CHO/EphB6 (Fig. S5).

## 4 Discussion

Approximately 10 % of the protein kinases lack conserved amino acids in the kinase domain, which are required for its activation [49,50]. EphB6 has been identified as one of the RTKs without kinase activity [51]. However, EphB6 expresses numerous tissues and cells to maintain physiological homeostasis, including kidney [52], vascular smooth muscle [53], and T cells [38,54]. The high expression of EphB6 has been observed in the thymus, pancreas, and brain [55,56]. Many reports have demonstrated that EphB6 is fully functional, but its kinase activity is undetected. Human epidermal growth factor receptor 3 (HER3) is one of the most well-known pseudokinases, which belongs to the human epidermal growth factor receptor (EGFR) family [57,58]. HER3 lacks kinase activity but plays pivotal roles such as cell proliferation, physiological homeostasis, and tumor development by forming a heterodimer with human epidermal growth factor receptor 2 (HER2) or EGFR [59,60]. The clinical trial of patritumab deruxtecan (HER3-DXd), HER3 targeting antibody-drug conjugates, is ongoing in locally advanced or metastatic NSCLC with EGFR mutations [61]. Therefore, pseudokinases may become therapeutic targets for cancer. EphB6 has been noted the contribution of oncogenic role in various cancer types, including lung [62], colon [19,63], and breast cancers [64]. Interestingly, crosstalk between EphB6 and EGFR cooperates in cancer progression [65]. Another group suggests a possible interaction between EphB6 and HER2 by imaging-based analysis [66]. The interaction of HER2 with EphB6 activates a compensatory signaling response following treatment with pertuzumab, an inhibitor of HER2-HER3 heterodimerization. These findings provide further functional expansion for EphB6 and enhance the potential of EphB6 as a therapeutic target for cancer. Furthermore, tyrosine phosphorylation-independent regulation of EphA2 mediates tumor aggressiveness, including metastasis, invasion, and poor prognosis [67,68]. Kinase activity-independent regulation of Eph receptors might be an attractive target for cancer therapy [69]. In contrast, tumor suppressive functions of EphB6 have been proposed in various cancers. Diminishing expression of EphB6 leads to tumor malignancy [23,35,70]. Further investigation is necessary to clarify these opposing functions of EphB6 in cancer. The management by endocytosis may contribute to modulating the amount of Eph receptors and ephrin expression at the cell surface [71]. In that case, Eb_6_Mab-3 will contribute to the elucidation of the EphB6-related biological responses by detecting EphB6 with moderate to high affinity in flow cytometry (Fig. 2, Fig. 3, Fig. 4).

The clinical trials targeting EphB6 have not been confirmed to date. However, the development of specific antibodies such as Eb_6_Mab-3 is also desired to clarify the function of EphB6 in the basic research, diagnosis, and treatment. The expression of EphB6 is inversely related to the expression of the molecules necessary for antitumor immunity, such as chemokine receptors and MHC genes [24]. Eb_6_Mab-3 might be helpful for the analysis of the immunological cold tumor microenvironment.

Eb_6_Mab-3 is unsuitable for immunocytochemistry against paraffin-embedded sections of cells (Fig. S5), suggesting that the Eb_6_Mab-3 recognizes a structural epitope of EphB6. Furthermore, Eb_6_Mab-3 exhibited different affinity to exogenously and endogenously expressed EphB6 (Fig. 4). The protein folding or modification may be different in both cell lines, which influences the affinity of Eb_6_Mab-3. Therefore, the epitope analysis is essential to answer the questions. We will determine the epitope of Eb_6_Mab-3 in a cell-based analysis such as PA tag-substituted analysis (PA scanning) and RIEDL insertion for epitope mapping (REMAP) method [72,73].

We have previously elevated antibody-dependent cellular cytotoxicity (ADCC) and complement-dependent cytotoxicity activities by switching isotypes and performing defucosylation in mAbs [[74], [75], [76], [77]]. Since Eb_6_Mab-3 is mouse IgG_1_, which lacks ADCC activity, it will be converted to a mouse IgG_2a_ version to examine the efficacy of antitumor effects in tumor xenograft models in future studies.

## CRediT authorship contribution statement

**Tomohiro Tanaka:** Writing – original draft, Investigation, Funding acquisition. **Yu Kaneko:** Investigation. **Haruto Yamamoto:** Investigation. **Guanjie Li:** Investigation. **Shiori Fujisawa:** Investigation. **Hiroyuki Satofuka:** Investigation, Funding acquisition. **Keisuke Shinoda:** Investigation. **Takuya Nakamura:** Investigation. **Mika K. Kaneko:** Conceptualization. **Hiroyuki Suzuki:** Writing – review & editing, Funding acquisition. **Yukinari Kato:** Writing – review & editing, Project administration, Funding acquisition, Conceptualization.

## Author disclosure statement

The authors have no conflict of interest.

## Funding information

This research was supported in part by Japan Agency for Medical Research and Development (AMED) under Grant Numbers: JP24am0521010 (to Y.Kato), JP24ama121008 (to Y.Kato), JP24ama221339 (to Y.Kato), JP24bm1123027 (to Y.Kato), and JP24ck0106730 (to Y.Kato), and by the Japan Society for the Promotion of Science (JSPS) Grants-in-Aid for Scientific Research (KAKENHI) grant nos. 22K06995 (to H.Suzuki), 24K18268 (to T.T.), 24K11652 (to H.Satofuka), and 22K07224 (to Y.Kato).

## Declaration of competing interest

The authors declare the following financial interests/personal relationships which may be considered as potential competing interests:

Yukinari Kato reports financial support was provided by 10.13039/100009619Japan Agency for Medical Research and Development. Yukinari Kato, Hiroyuki Suzuki reports financial support was provided by 10.13039/501100001691Japan Society for the Promotion of Science. Hiroyuki Satofuka, Tomohiro Tanaka, reports financial support was provided by 10.13039/501100001691Japan Society for the Promotion of Science. If there are other authors, they declare that they have no known competing financial interests or personal relationships that could have appeared to influence the work reported in this paper.

## Figures and Tables

**figs1:**