# Supplementary material for: Development of a novel anti-erythropoietin-producing hepatocellular receptor B6 monoclonal antibody Eb6Mab-3 for flow cytometry
Source: Biochem Biophys Rep. 2025 Feb 21;41:101960. doi: 10.1016/j.bbrep.2025.101960 (PMC11891606; doi:10.1016/j.bbrep.2025.101960)
Supplement: Multimedia component 1 [file mmc1.docx]

**
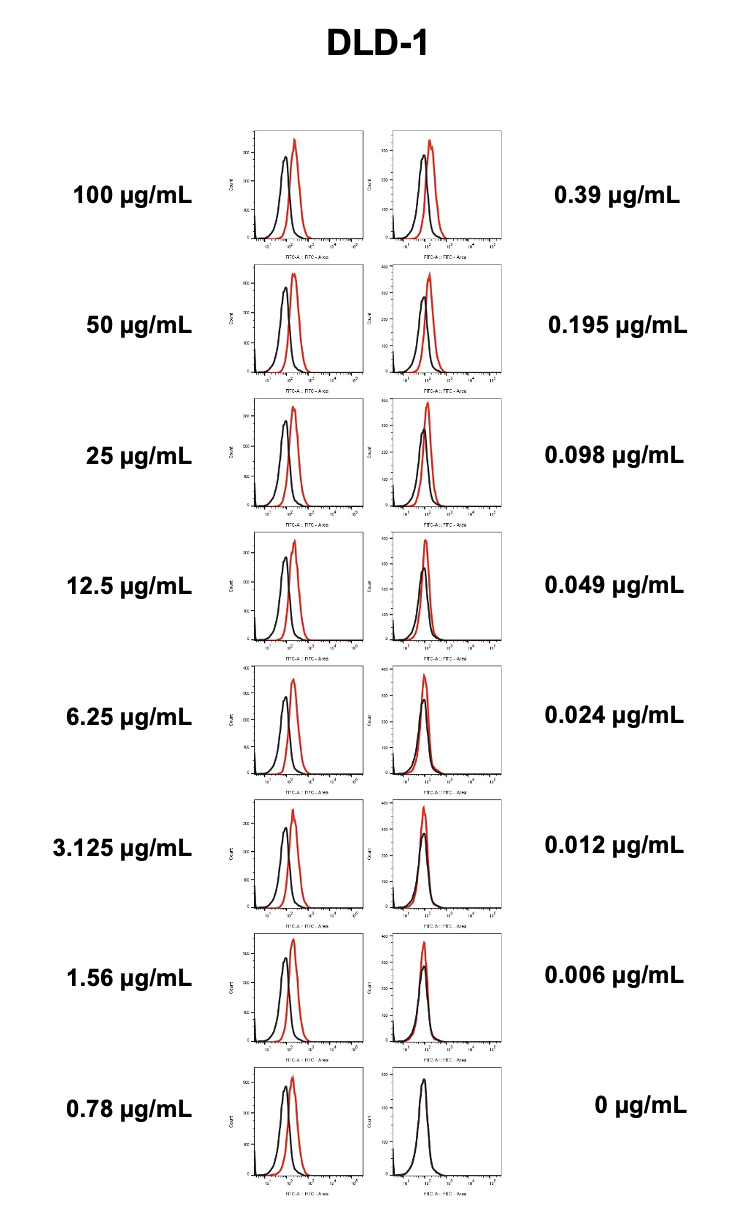
**

**Fig. S1 Flow cytometric analysis of anti-EphB6 mAbs.** DLD-1 cells were treated with 0.006–100 µg/mL of Eb_6_Mab-3, followed by treatment with Alexa Fluor 488-conjugated anti-mouse IgG. Fluorescence data were collected using the SA3800 Cell Analyzer. Black line, control (no primary antibody treatment).

**
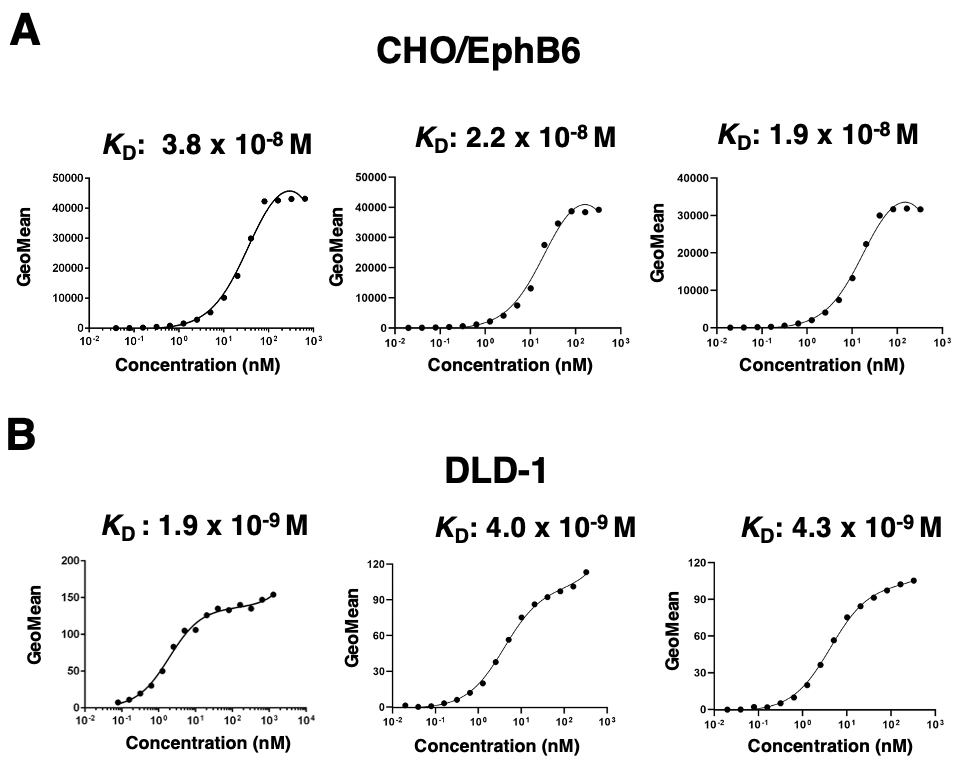
**

**Fig. S2 Determination of the binding affinity of Eb_6_Mab-3.** CHO/EphB6 (A) and DLD-1 (B) cells were suspended in 100 µL of serially diluted 50 µg/mL to 0.003 µg/mL of Eb_6_Mab-3 for CHO/EphB6, 100 µg/mL to 0.006 µg/mL of Eb_6_Mab-3 for DLD-1. Then, cells were treated with Alexa Fluor 488-conjugated anti-mouse IgG. Subsequently, the geometric mean values from fluorescence data were determined using the SA3800 Cell Analyzer. The three independent measurements were shown. The *K*_D_ values were calculated by GraphPad PRISM 6 software.

**
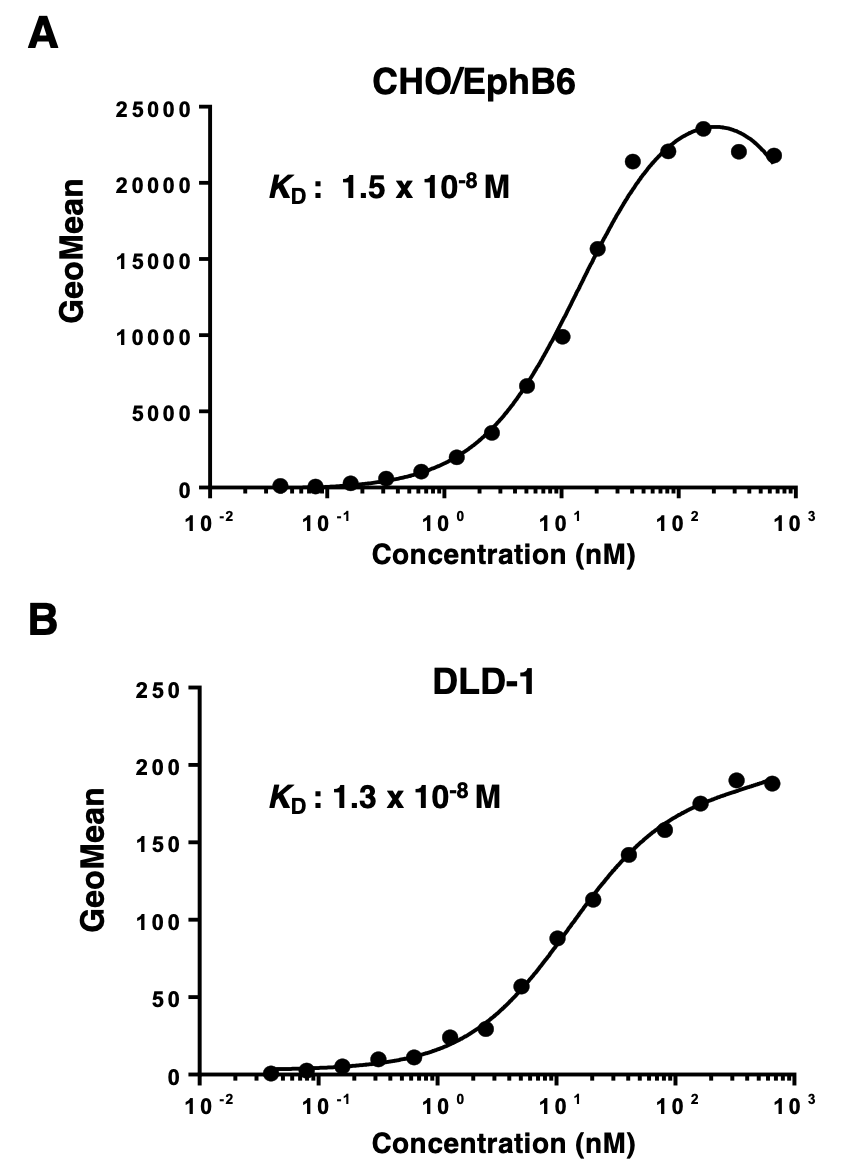
**

**Fig. S3 Determination of the binding affinity of T49-25.** CHO/EphB6 (A) and DLD-1 (B) cells were suspended in 100 µL of serially diluted 50 µg/mL to 0.003 µg/mL of T49-25. Then, cells were treated with Alexa Fluor 488-conjugated anti-mouse IgG. Subsequently, the geometric mean values from fluorescence data were collected using the SA3800 Cell Analyzer, following the calculation of the *K*_D_ by GraphPad PRISM 6 software.


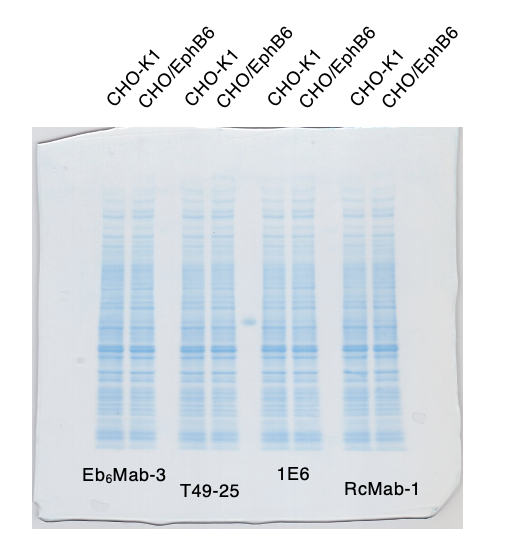


**Fig. S4** A polyacrylamide gel after transfer was stained by Bio-Safe CBB G-250 (Bio-Rad Laboratories, Inc., Berkeley, CA, USA).

**
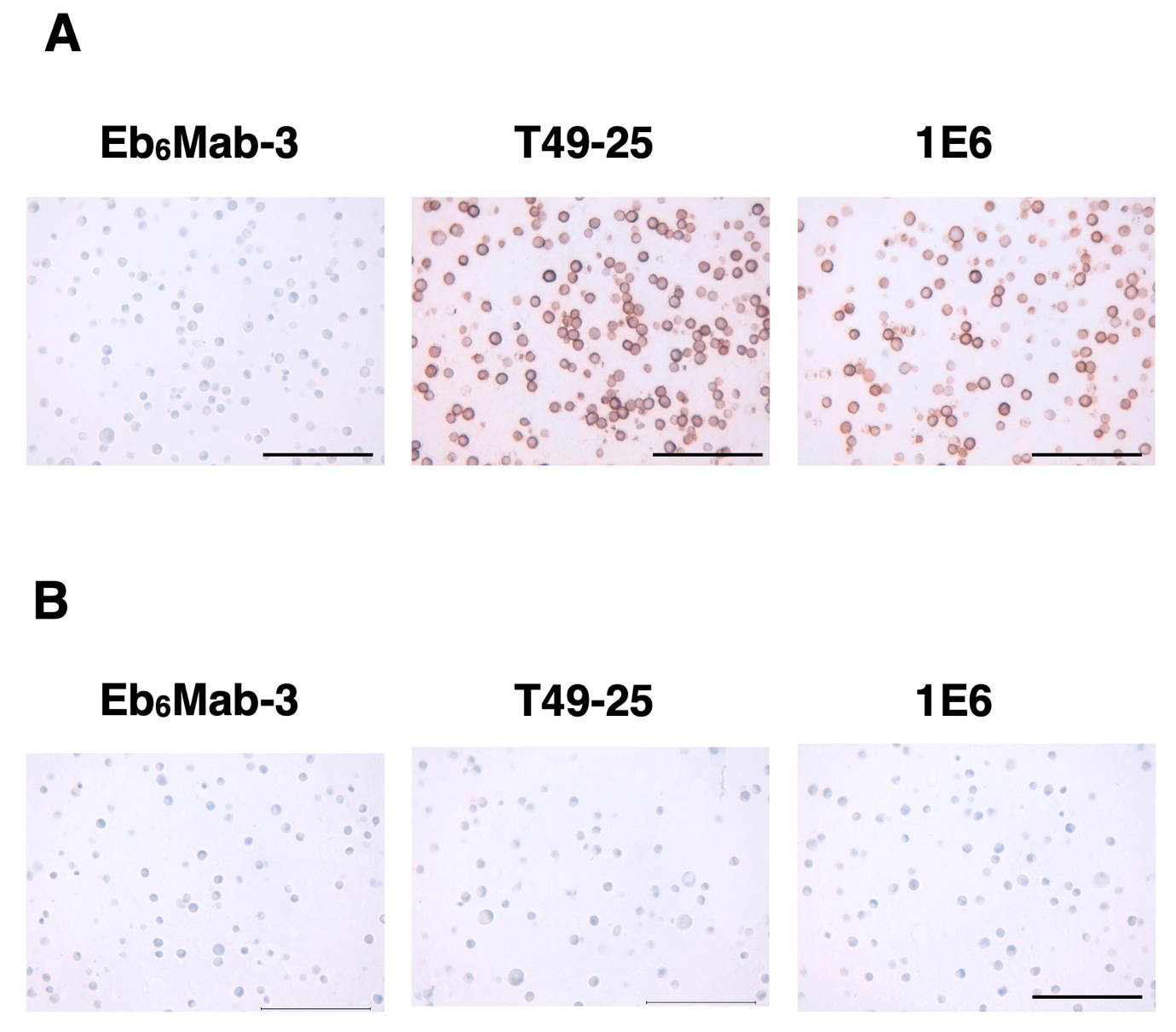
**

**Fig. S5.** CHO/EphB6 **(A)** and CHO-K1 **(B)** cell blocks were prepared using iPGell (Genostaff Co., Ltd., Tokyo, Japan) and fixed with 4% paraformaldehyde phosphate buffer solution (FUJIFILM Wako Pure Chemical Corporation). The blocks were processed to make 4 μm thick paraffin-embedded cell sections that were directly autoclaved in a citrate buffer (pH 6.0; Nichirei Biosciences, Inc., Tokyo, Japan) for 20 min. These sections were blocked using the SuperBlock T20 Blocking Buffer (Thermo Fisher Scientific Inc.), and then incubated with Eb_6_Mab-3 (5 μg/mL), T49-25 (5 μg/mL), or 1E6 (an anti-DYKDDDK mAb, 5 μg/mL) for 1 hr at the room temperature. The sections were then treated with the Envision + Kit (Agilent Technologies Inc.) for 30 min. Color was developed using 3,3’-diaminobenzidine tetrahydrochloride (DAB; Agilent Technologies Inc.), followed by counterstaining using hematoxylin (Merck KGaA). Scale bar, 100 μm
